# ‘I’m looking for support, solidarity, and anecdotes from anyone who has been through something similar’: A content analysis of parents seeking or sharing advice on breastfeeding via Reddit

**DOI:** 10.1177/13591053251318475

**Published:** 2025-02-24

**Authors:** Julia Yates, Kimberley T Jackson, Richard Booth

**Affiliations:** The University of Western Ontario, Canada

**Keywords:** advice, breastfeeding, parenting, Reddit, social media, support

## Abstract

Parents are increasingly turning to social media for breastfeeding-related information. Reddit, which allows users to anonymously discuss with individuals around the world, has recently increased in popularity among parents. Given breastfeeding’s importance, it is imperative to examine the behaviours of information-seeking parents to ascertain what information is being sought out and shared. The purpose of this study was to explore parents’ use of Reddit to seek out and share breastfeeding-related information. The Naturalistic Inquiry method was employed to explore data extracted from the breastfeeding SubReddit (i.e. a forum of a particular topic where users can post/discuss the topic; /r/breastfeeding). Via thematic analysis, four themes emerged: (1) breastfeeding-related challenges; (2) ‘normal’ behaviours for age/development of infant; (3) weaning; and (4) returning to work. An increased understanding of the factors involved in parents’ decisions to seek support online, rather than from professionals, may provide important insights into breastfeeding support gaps.

## Introduction

The 21st Century has been defined as ‘the digital age’- a time when copious amounts of information are widely available to people through technology (Cambridge Dictionary, 2024). With each year, technology becomes further embedded into human lives, with the use of social media being one of the most popular online activities. In January 2024, approximately 5.04 billion people around the world were using social media – 266 million of which came online within the last year (Kemp, 2024). Reasons for using social media are varied and plentiful, such as staying connected with friends and family, filling spare time and reading news stories (Dixon, 2024). Increasingly, social media platforms have also been used to spread and seek health-related information (Chen and Wang, 2021). One advantage of using social media in this way is that it allows users across the world to both receive and provide health information and knowledge within seconds (Bennett and Glasglow, 2009); however, health information found on social media may also expose users to inherent risks. One of the main limitations of online health information is a lack of quality and reliability caused by authors being unknown, or the information being unreferenced, incomplete, or incorrect (Moorhead et al., 2013; Pirraglia and Kravitz, 2013). When users are unaware of these risks, it may lead to an over-reliance on easily accessible, but inaccurate information (Ventola, 2014).

Health-related information seeking has been increasingly documented as a critical coping strategy in health-promotion activities and psychosocial adjustment to health changes (Lambert and Loiselle, 2007). Defined as a deliberate attempt to meet a health information need (Johnson, 1997), health information-seeking behaviour has rapidly evolved with the emergence and growing popularity of social media platforms (Bratland et al., 2024; Dutta-Bergman, 2004). Online health information-seeking behaviour may have a positive influence on health information consumers since they are more likely to better adhere to treatment after obtaining adequate information on their health condition (Powell et al., 2011). Therefore, research is needed on adults’ online health information-seeking behaviours to identify gaps in health knowledge, improve health literacy and promote health equity. In doing so, we may be able to inform best practices for health communication and intervention strategies (Bratland et al., 2024).

One group of individuals who are increasingly turning to social media for health-related information and support is parents of young children. In a study conducted by Today’s Parent (2018), data collected from over 1000 parents in Canada with young children found that approximately 60% of parents were using social media platforms to find parenting information. Further, in a nationally representative report by the C.S. Mott Children’s Hospital National Poll on Children’s Health (2023; *n* = 614), it was found that most mothers of young children use social media to look for parenting advice (84%) or to share their experiences (63%). Interestingly, among the topics that parents reported using social media to learn about, breastfeeding was a common topic of interest for almost 40% of parents (C.S. Mott Children’s Hospital, 2023). A recent study by Skelton et al. (2018) explored the effects of social media usage by mothers (*n* = 33) in the United States on breastfeeding outcomes. Participants in this study reported that social media provided a sense of community support around breastfeeding, normalized breastfeeding, empowered breastfeeding and allowed mothers to share their experiences around breastfeeding (Skelton et al., 2018). Finally, a Canadian study which examined digital technology use in the transition to parenting, identified that women often used digital technologies to seek information about their pregnancy, postpartum and newborn health. In particular, sites such as The Milk Meg (themilkmeg.com) and Dr. Jack Newman’s ‘International Breastfeeding Centre’ (https://ibconline.ca) were cited as commonly used for information and to assure mothers their experiences were normal (Donelle et al., 2021). While mothers frequently used technology for information seeking, connecting with other supports, and tracking of different perinatal and parenting factors, they also acknowledged that they had concerns about the credibility and trustworthiness of online resources (Donelle et al., 2021). However, currently, the quality and accuracy of information in this context remains unknown, which may lead to misinformation regarding breastfeeding being widely disseminated.

Research highlights how essential both formal and informal social supports are to improving breastfeeding outcomes, including both the initiation and duration of breastfeeding (Brockway et al., 2017; Davidson and Ollerton, 2020). Social support involves people seeking support from their social networks and relational partners in the face of stressors. The support provided varies from ‘just listening’ to providing emotional, informational and/or instrumental help (Bandura, 1986). For example, Baño-Piñero et al. (2018) found that participants who communicated their breastfeeding challenges and doubts within breastfeeding social support networks were more likely to breastfed longer compared to participants who did not have informal social support available. In terms of breastfeeding informal social support, much of the literature has focussed on support received from one’s intimate partner, with strong evidence suggesting that a woman’s intimate partner is an important contributor to breastfeeding success (Dennis, 1999). However, a knowledge gap exists regarding how breastfeeding women seek and utilize informal social supports, such as those received from social media networks, as a means of breastfeeding support.

Several theories may contribute to our understanding of behaviours, beliefs, and attitudes of family and friends that can influence the success (or lack thereof) of achieving one’s infant feeding goals. For example, Bandura’s (1986) Social Learning Theory considers environmental and cognitive factors which interact to influence human behaviour and learning and underscores the importance of observing, imitating and modelling the behaviour of others around us. This theory was extended to the breastfeeding context by Dennis (1999) whereby breastfeeding self-efficacy and breastfeeding outcomes can be enhanced via vicarious experiences, role models and verbal persuasion (among other factors). Alternatively, Social Support Theory (Barnes, 1969; Cassel, 1976; House et al., 1988), describes how a person’s social connections can provide emotional, informational, and practical support to help them cope with stressful or challenging experiences, which is often the case among breastfeeding individuals. Together, these theories give credence to the importance and possible impact of social networks on a woman’s decision, capacity and potential success to breastfeed.

While YouTube, Facebook and Instagram are widely used online platforms among U.S adults, the use of the Reddit platform has been steadily increasing, with 22% of adults reporting using Reddit in 2023 compared to only 11% in 2019 (Pew Research Centre, 2023). Reddit is a publicly available platform that is used to stimulate discussion from participants around the world with access to the internet and an electronic device (Reddit, 2016). On Reddit, various forms of media (e.g. text, images, videos) are shared by participants in topic-specific sections (i.e. SubReddits) wherein participants can view posts within conversation threads, respond to posts and ‘upvote’ or ‘downvote’ posts (Record et al., 2018). The breastfeeding Subreddit (i.e. /r/breastfeeding) is a self-proclaimed ‘community to encourage, support, and educate parents nursing babies/children through their breastfeeding journey’ with nearly 150 thousand members. Marcon et al. (2021) suggested that the anonymity afforded to individuals using Reddit plays a central role in strengthening the quality of conversations as people are not restrained by the fear that would typically come with aligning their posts with their true identities, as is the case on many other social media sites. As such, Reddit presents the opportune platform that allows participants to anonymously solicit information and freely converse online, making this an ideal platform for parents to seek judgement-free advice related to breastfeeding.

Despite the large amount of publicly available breastfeeding-related information, parents are still choosing to seek advice from others via social media platforms. Given the importance of breastfeeding for infants and mothers (Dieterich et al., 2013), it is imperative to ensure that accurate breastfeeding information is being shared. To this end, it is critical to examine the behaviours of such parents to ascertain what information is being sought out to improve our current dissemination practices while also ensuring that harmful misinformation is labelled as such. Based on the specific qualities of the Reddit platform (e.g. anonymous posting), and the large number of members of the breastfeeding SubReddit (i.e. 150 K), it is likely that this is a common platform that many parents are using to seek out and share breastfeeding-related information. Despite this fact, to the authors’ knowledge, there has been no prior exploration of the use of this platform in relation to breastfeeding behaviours. As such, the purpose of this study was to explore parents’ use of Reddit to seek out and share breastfeeding-related information. By focussing on the seeking and sharing of breastfeeding-related information, we hope to contribute to a growing body of literature around breastfeeding supports, including what information people are most interested in, what breastfeeding areas are currently under supported, and how the online, anonymous nature of Reddit may be a suitable medium to pursue when developing new breastfeeding supports.

## Methods

### Study design

Per Lincoln and Guba (1985), the Naturalistic Inquiry method was employed to explore parents’ use of Reddit to seek out and share breastfeeding-related information with the goal of gathering a thick description from the online data. In line with Lincoln and Guba’s (1985) trustworthiness concepts of naturalistic research, methods to uphold credibility, transferability, dependability and neutrality were employed. Specifically, credibility was upheld through transparency of methods, transferability through the presentation of findings that may have applicability within other settings where parents share/seek advice related to breastfeeding, dependability through the presentation of consistent findings that represent a specific moment in time, and neutrality through self-reflection and the keeping of reflexive notes throughout the research process.

As data on Reddit is in the public domain, individuals who post information onto this platform have no reasonable expectation of privacy. As such, no ethical approval was required for this study. Further, any identifying information provided by participants and/or Reddit accounts were anonymized during this study to preserve anonymity.

### Data collection

Using a purposive, systematic qualitative data collection method, a subset of publicly available posts, comments and questions on the breastfeeding SubReddit published during January and February 2024 were examined (*N* = 997). Each post or comment served as a unique unit of qualitative data. To extract data from the SubReddit, RStudio was utilized, following which the data was uploaded into NVivo 14 qualitative analysis software (Lumivero, 2024).

A singular file containing the top content posted on the breastfeeding SubReddit between January and February 2024 was produced (i.e. ~1000 discussions and responses). Discussion threads were imported into NVivo 14 (Lumivero, 2024), inclusive of all data from each thread (i.e. date of post, username of poster, comments, upvotes and downvotes). According to Reddit, if a user believes that something contributes to a conversation, then that user can upvote it. Conversely, if the user things it does not contribute to the SubReddit it is posting in and/or it is off topic, the user can downvote it (Reddit, 2023). Further organization of the data was required prior to analysis given the hierarchical structure of Reddit posts. As such, conversation threads from the search queries were organized by *Top* comments, which showed comments that had been interacted with the most, through upvoting and downvoting. Threads with rich and thick narratives were purposively selected for study (e.g. those threads with minimal, confusing or spam posts were removed). After all data was extracted, the conversation threads were imported into NVivo 14 for content analysis.

### Data analysis

An inductive, semantic thematic analysis (Braun and Clarke, 2006) was employed with the goal of categorizing large quantities of text into concise groupings/themes with shared meanings. Given the nature of the data units for this study being limited to what was shared on the SubReddit, semantic thematic analysis allowed the authors to identify themes using the explicit meaning of the data as we were unable to capture participants’ assumptions that are required for latent analysis. In line with this approach to analysis, the objectivism epistemological view was taken, which purports that meaning exists within an object (in this case, a singular post on Reddit) independent of the subject (in this case, the person posting on Reddit). Given the anonymous nature of Reddit and that we were unable to ascertain any details on participants, this epistemology positioned us to create knowledge using the data that we had access to.

To conduct this analysis, NVivo 14 was used to code content categories within the data and to help link these patterns together in the text to make deeper connections. First, the research team familiarized themselves with the data and to develop a preliminary coding framework by reading and re-reading the data and noting down any initial ideas/assumptions that came up during these readings. Next, once the research team discussed our initial ideas, preliminary codes emerged based on meaningful groupings and were documented in NVivo 14 by placing the posts of each Reddit thread into nodes based on common themes. Potential categories were then created using an inductive thematic analysis method wherein the researcher reading the threads developed category names that best described their overarching message. The research team then met again to discuss the entire data set and to generate a thematic map of the overarching analysis. Once refinements were made, the researchers felt the themes accurately represented the dataset, and consensus was reached between the research team, in-depth analysis was completed wherein clear definitions and names for each theme were developed. When producing the final manuscript, the research team selected the most vivid, compelling pieces of data to represent each theme.

## Results

Following data analysis, four themes and three subthemes emerged regarding parents’ use of Reddit to seek out and share breastfeeding-related information, including: (1) breastfeeding-related challenges (subthemes: pain – will it ever end?, sleep – seeking solidarity, supply – this is not sustainable); (2) ‘normal’ behaviours for age/development of infant; (3) weaning – seeking independence; and (4) returning to work – transition preparations. An overarching conclusion of analysis was that users went to Reddit looking to feel less alone in their experiences, with one participant exemplifying this by sharing ‘I’m looking for support, solidarity, and anecdotes from anyone who has been through something similar’.

### Breastfeeding-related challenges

Within the breastfeeding SubReddit, a common theme of discussion was challenges related to breastfeeding. These challenges broadly included those related to pain (~32.5% of posts), milk supply (~12.5% of posts) and sleep (~12.5% of posts).

#### Pain- will it ever end?

Breastfeeding-related pain was typically described by self-reported mothers as relating to the act of breastfeeding. In most cases, women experiencing pain were seeking advice on how to alleviate the pain and/or seeking out others who had experienced similar pain to feel less alone. One participant shared:

I am STRUGGLING breastfeeding. I know how important it is for baby but I HATE IT. Been going 4 months and I am TRYING to stick with it until at least 6 months. I just miss my boobs not hurting all the time. I miss sleeping GOOD. I can’t sleep on my sides or I get clogged ducts. I miss being able to run and workout without my boobs hurting. I can only wear like 3 super loose bras I own. I feel like it’s so hard to cuddle my baby cuz my boobs hurt, especially as my baby is starting to weigh more… I just want my body back… Does anyone else feel this way!? How did you keep going?

Many users shared similar sentiments to this participant, expressing the immense toll that breastfeeding has taken on their bodies, with many not knowing how they are supposed to carry on. Despite most participants sharing that they had consulted with various sources of support (e.g. lactation consultants, health professionals, other breastfeeding mothers), none of the suggestions seemed to alleviate the pain and frustration, thereby contributing to mental health challenges and causing mothers to reach out to the SubReddit for any other suggestions. One mother shared this sentiment:

I am breastfeeding my 10 weeks old baby, but I don’t know if I can do this anymore. I am so tired of being frustrated and in pain because of the bad latch he has.. he hates to latch correctly, whenever he does latch correctly he gets so mad and starts crying. And I feel this is a bit too much for me, I’ve fixed both his tongue tie and lip tie, I’ve seen 3 lactation consultants, I’ve done breastfeeding exercises for his mouth and I am struggling so hard. And so is he. Did anyone go through this and managed to resolve this? I don’t know what to do anymore and what to try, my breasts are so sore (I am using creams and medicine) and I am so desperate at this point that I feel that it’s taking a big toll on my mental health :(

For some mothers, the desire to stop breastfeeding due to the pain left them feeling guilty, with one expressing that ‘The only issue is I have this feeling like I am letting my baby down. It feels like I’m being selfish by wanting to stop already due to pain’. Nearly every post related to pain concluded with users asking if anybody else had experienced this pain or if anyone had any suggestions on how to alleviate their pain.

#### Milk supply – seeking solidarity

For those users who posted about perceived challenges with milk supply, most posed questions relating to if their experiences were ‘normal’. For example, one participant posted a question about the normalcy of a discrepancy in their milk supply between breasts, asking ‘I can usually get about 2–2.5 oz out of the left while only getting about one out of the right. Is this normal? Do I need to be trying to get more out of my right breast?’. Most comments replying to posts of this nature included encouraging messages of support and assurances that these experiences were completely normal and happened to many breastfeeding mothers. Additionally, many mothers shared concerns when they noticed their supplies dropping, with some noting the added layer of financial concerns if their frozen/stored breast milk were to run out. One mother shared:

Last week I noticed frustration at the breast, arching back, pulling away from nipple, nursing, crying, nursing crying, getting next to NOTHING having to flip flop between both sides to make her happy. Feeling confused… Sad. I’ve dipped into some of my freezer stash and I’m scared I’m losing my supply. I can’t afford to go buy an expensive pump. What do I do?

Other mothers shared concerns with the price of formula/nutritional supplements if their supply issues were to continue. Some responses to these concerns shared resources that women could reach out to for nutritional supplies; however, the geographic differences in users of this SubReddit often proved as barriers to accessing the resources being shared.

#### Sleep – this is not sustainable

For many users, breastfeeding contributed to sleep challenges both for parents and infants. In most cases, the sleep disturbances were due to the limited time between overnight feeds for their infants. One participant shared:

I don’t know how long I can sustain this. My 9 week old will not go any longer than generally an hour to an hour and a half between feeds… The only time we get a longer stretch is when my husband gives him a bottle at night so I can get a bit of extra sleep but last night he fed every 40 minutes to an hour overnight and I had also fed him every hour to hour and a half during the day. I really really want breastfeeding to work out but I feel so stuck…

The lack of sustainability of feeding schedules, which contributed to sleep disturbances for most mothers, was shared by many. Similarly, many mothers posed breastfeeding-related questions around how to get their children to sleep longer. For example, one user asked ‘Should I just shorten the middle of the night feed to feeding on both sides once and top up with a bottle? Will that make him sleep longer?.”

Outside of the issue of infants waking up to feed, mothers also shared their troubles with sleeping through the night due to being awoken by the pain of having full breasts. One mother shared that “[The infant] has just begun sleeping through the night however I literally cannot because I wake up at some point in pain with being soooo full and rock hard. How does one manage this? Advice appreciated on options!!!’. After sharing their concerns, many users also commented on what actions they are currently taking in the hopes that others will have suggestions they have yet to try. One participant asked:

What the heck am I doing wrong. I tried no flow tea, cooling gel pads, cabbage leaves & the only thing that kind of helps is block feeding but how the heck do you do that at night when he isn’t consistent about wake-up time. Desperate here for any other advice.

Usually, other users responded in these cases with advice on what worked for them or for other people they knew.

### ‘Normal’ behaviours for age/development of infant

Another common subject of discussion on the breastfeeding SubReddit were questions related to if infant behaviour was considered to be ‘normal’ for their current age/level of development, according to the other users (~15% of posts). For example, one participant asked ‘She has spent this morning nursing, drifting off, catching a quick nap, waking up to play a little, rinse and repeat. Is this behaviour normal for her age?’. While most responses to these questions confirmed that these behaviours were ‘normal’, some expressed concerns if these behaviours had never been mirrored in their own children. Beyond questions relating to infant feeding patterns, users shared specific concerns around discrepancies in what different sources were considering ‘normal’. Most commonly, what a mother was reading would not align with what their health practitioner was recommending. One mother explained:

I’m so confused and frustrated after my son’s 4 month well check. He only gained 1.08 lbs between 2 and 4 months. Dropped to 4th percentile. Everything the NP at the paediatrician’s office said in relation to his weight was the opposite of what I’ve read that data indicates… For the second time since his birth, the NP suggested not having enough calories in my diet produces ‘skim milk’. I’ve read that data shows mom’s diet doesn’t control the calorie or fat content of breast milk… Advice?

Discrepancies in information were also reported between different providers (e.g. nurse practitioner vs lactation consultant), which left mothers sharing feelings of confusion regarding which source to follow.

### Weaning – seeking independence

Many users posted to the breastfeeding Reddit hoping to ascertain advice on weaning practices (~12.5% of posts). Specifically, many mothers shared feelings of wanting or needing to wean their children off breastfeeding to gain some of their pre-breastfeeding freedom back. One mother shared the isolation of the breastfeeding experience, leading to the desire to wean her child off the practice:

I have a now to be soon 2yo and I’ve breastfed her all this time, it used to be only whenever she was hungry now it’s even if she gets a little ouch she comes and wants to be soothed with it, she needs it to go down for a nap or sleep for the night… Now I’m sure that it can’t go on like that forever right? It breaks my heart but I can’t even go out on a walk for her to fall asleep without it because she’ll cry until we’re home. So now I can’t go anywhere around her nap time I have to be home… it starts to drive me crazy and sometimes I’m overwhelmed, I can’t do anything alone or with my husband really and it’s just so isolating. So my initial question is, do they wean themselves off? Do I have to do it? And if so how?

Feelings of isolation related to breastfeeding were echoed by other participants who felt as though weaning their children off breastfeeding was the only way to gain back some of their independence.

Other reasons for users sharing their desire to wean their children off breastfeeding stemmed back to the pain of the experience. In these cases, many women were looking for advice on best practices when it comes to which feed to eliminate first, in the hopes that they could speed up the process. One user shared, ‘We’ve had a painful nursing journey and I’m ready to have my nipples feel okay again… If we night wean, will bedtime and morning get better? Where do I go from here?’. Whether it be related to freedom or pain from breastfeeding, most weaning-related posts were seeking advice from others on best practices in the hopes that their experiences may mirror those who commented.

### Returning to work – transition preparations

For mothers who chose to return to work while also breastfeeding their infants, posts to the SubReddit centred around how to regulate their supply with their changing schedule and/or how much breastmilk they should be sending to daycare services (~17.5% of posts). Many users shared confusions regarding how returning to work, and therefore how altering their regular pumping/feeding schedule, would impact their milk supply. One mother asked, ‘So how does BF work when I go back to work… Is my only option to pump and be over supplied? Like just add one pump in a day?’. Conversely, other mothers shared concerns around their new tentative pumping schedules, as a result of returning to work, decreasing their supply. One mother shared this sentiment, ‘I am wondering if I can get away with only pumping twice during an 8 hour workday since I feed one side at a time and would pump both sides… Might this work or will this drop my supply?’.

For those users who were sending their children to daycare when they returned to work, questions generally stemmed around how to prepare for the transition to daycare, how much breastmilk to send with their infants or other users’ experiences of supplementing their infants diets with substances other than breastmilk. One user shared:

I’m expecting to go back to work in about 3 weeks, so I’m trying to pump in order to get a stash for daycare. After feeding I usually pump for about 15–20 minutes. I can only get at most 1 oz from each side. Is this normal? If not, what can I do to increase production?

Other users echoed this sentiment, asking the best ways to prepare for their infants to attend daycare. Similarly, many women asked follow-up questions related to the exact amounts of breastmilk other users had sent with their infants when they initially started attending daycare. For example, one user asked, ‘How many bottles and ounces did you prepare for your 4 month old?’.

Beyond these logistical questions related to breastmilk at daycare, some participants were also seeking out other users’ experiences of supplementing their infants’ diets with substances other than breastmilk. Usually, these queries were accompanied by concerns around introducing formula feeding and complimentary foods for their infants, such as the introduction of new foods (e.g. purees). One mother shared her experience of this:

Baby was exclusively breastfed until I returned to work at 12 weeks. I pump at work and bf at home. I pump 13–16 ounces during the workday while I am away from him for ~10 hours. Baby is gaining weight well per paediatrician. I have no freezer stash. He is 5 months and we have been introducing purées. Daycare has been requesting that I send more milk as the baby gets upset after his bottles are finished. They feed him 4 oz every 3–4 hours. I have tried pumping more frequently which just produces the same net amount at the end of the day. My husband feels we should send formula to daycare to supplement but I am worried they will just feed him excessive amounts of formula to pacify him and my supply will start to dwindle. Should I send formula to daycare in addition to my pumped milk?

Other users shared similar sentiments to this post, expressing frustrations with daycare requesting additional milk that their supply could not keep up with, and therefore feeling as though the only solution was to initiate formula feeding.

## Discussion

The purpose of this study was to explore parents’ use of Reddit to seek out and share breastfeeding-related information. Findings from this study highlight the various topics that parents tend to seek breastfeeding-related advice about, including challenges related to pain, supply and sleep. Further, parents frequently asked questions pertaining to whether their infants were displaying ‘normal’ behaviours for their age, weaning their children off breastfeeding and the implications on breastfeeding when returning to work.

Breastfeeding-related challenges are highlighted throughout literature, with pain related to breastfeeding being the most cited challenge for mothers (Babakazo et al., 2022; Zimmerman et al., 2022). A Canadian study on the measurement of breastfeeding-related pain reported that pain during the early days of breastfeeding is common, is often experienced as distressing and severe, and can lead to breastfeeding cessation (Jackson et al., 2019a). Unfortunately, breastfeeding-related pain is not commonly discussed in perinatal contexts, nor literature. In fact, a qualitative study exploring breastfeeding-related pain found that, overwhelmingly, women were surprised at their pain, were not aware this type of pain existed before they had birth, and wished that they knew about it so they could have been better prepared to manage it (Jackson et al., 2019b). Where discourse does exist in the realm of breastfeeding-related pain, there remains a great deal of ‘sugar coated’ information related to the realities of breastfeeding pain available from reputable sources. For example, the Centre for Disease Control and Prevention states that ‘Although your breasts and nipples may be tender or uncomfortable, once your baby is well-latched, breastfeeding should not be painful’. (CDC, 2024, pp. 6). Interestingly, the impact of this lack of support and transparency from professional organizations was noted in a recent study by Regan and Brown (2019) who explored the experiences of women (*n* = 14) using online supports for breastfeeding in the United Kingdom via semi-structured interviews. Mothers in this study shared that they were drawn to online support due to a lack of effective professional support related to the physiology and management of breastfeeding (Regan and Brown, 2019). Via online groups, these mothers felt reassured and were greeted with around the clock empathy that they noted as being less daunting than traditional in-person support groups, with many even attributing their continuation of breastfeeding to this support they received (Regan and Brown, 2019). This finding is of critical importance as Bridges et al. (2018) noted that the most common topics that women were seeking online support for correlated with the most common breastfeeding challenges, which lead to early cessation. Combined, it is clear that support related to common breastfeeding challenges, as users in this study were seeking, can contribute to the continuation of breastfeeding. Future research should explore the best avenues for women seeking this support, as well as best practices for online support groups, so that healthcare professionals can direct women seeking support to these online forums in the hopes of promoting breastfeeding continuation.

A recent systematic review by Morse and Brown (2022) explored the benefits, challenges and impacts of accessing social media group support for breastfeeding. The most common concern in relation to social media breastfeeding support was the reliability of information being shared, which is a concern that has been echoed in the broader literature (Ellis and Roberts, 2019; Morse and Brown, 2022). Interestingly, in the current study, users on the breastfeeding SubReddit shared discrepancies in what different sources were considering ‘normal’ for development, including differences between their own research and various healthcare professionals. While no posts were found in the SubReddit from users sharing that the information in the group was more valuable than that from professionals, past research has found this to be true in some cases. In a study by Skelton et al. (2018), women who belonged to online breastfeeding support communities found that real-time information from individuals with lived experiences was valid and reliable, and shared feelings of trusting this information over professional advice. Together, while it is evident that individuals seeking support via online forums generally trust the information they are being provided, less is known about the reliability of said information. Future research would benefit from exploring the utility of online breastfeeding support groups as a source of reliable information.

A common thread throughout discussions on the breastfeeding SubReddit was women seeking support from those who had been through a similar experience, with the hopes of making them feel less alone in their challenges. Similar findings were echoed by Chivers et al. (2021) who explored the conversations of new mothers on an online parenting forum. Most frequently, mothers on this forum reached out when asking how to manage something, when seeking out someone with a similar experience, or to ask others if a problem they were experiencing was ‘normal’ in an attempt to normalize their own experiences or to confirm a problem (Chivers et al., 2021). Interestingly, while this parenting forum was not specific to breastfeeding, nearly 20% of the topics related to infant feeding, including milk supply (~4.6% of posts), feeding routines (~2% of posts), the hunger-sleep relationship (~1% of posts) and weaning (~0.5% of posts; Chivers et al., 2021) – all of which were also found to be common topics discussed on the breastfeeding SubReddit. The desire for their experiences to be validated and deemed ‘normal’ by their peers on online forums reflects a deeper issue of isolation among mothers who are breastfeeding. Healthcare professionals must take this information into consideration when providing breastfeeding-related supports. Future breastfeeding programmes must be cognizant of these feelings of isolation to ensure that supports are being provided for maternal mental health during the breastfeeding period.

The anonymous nature of Reddit may shed light on the behaviours of users. Specifically, the anonymity promised by Reddit may result in decreasing inhibitions and increasing self-disclosures – a behaviour that has been coined the ‘online disinhibition effect’ (Suler, 2004). Under the anonymous conditions of Reddit, people may self-disclose more intimate information because there are few, if any, related risks (Bargh et al., 2002). Based on this, the information being shared via the breastfeeding SubReddit may be particularly vulnerable and truthful compared to that which might be shared on a platform necessitating self-disclosure. The current study sheds light on this important phenomenon that can be applied to the breastfeeding sphere wherein lessons learned could be applied to future support groups for breastfeeding individuals.

### Limitations

The results of this study should be considered within the context of the study’s limitations. First, there is no way to ascertain the demographic characteristics of Reddit users. As such, there is no way to definitively conclude that users in the breastfeeding SubReddit were truly mothers who were breastfeeding. Second, due to the amount of data housed on the breastfeeding SubReddit, purposeful sampling of specific threads for analysis was undertaken. Specifically, only the ‘top’ threads from extraction period (i.e. inclusive of January-February 2024) that were deemed sufficiently thick in description were included in analysis. As such, this may bias the findings of this study. Finally, given the nature of the study and the inherent external validity limitations, the transferability of this study is lacking. Future research would benefit from exploring the sharing and seeking of breastfeeding-related information on other social media platforms (anonymous and public) to examine this phenomenon more broadly.

### Conclusion

The findings from this study are the first to explore parents’ use of Reddit to seek out and share breastfeeding-related information. Given the importance of breastfeeding to both mothers and infants, further research is required to understand the complexity of mothers seeking support via online forums more clearly, including which mothers are most likely to do so and why. Further, an increased understanding of the factors involved in mothers’ decisions to seek support online, rather than from professionals, may provide important contextual information regarding how to improve clear gaps in the current supports being provided by healthcare professionals.

## References

[bibr1-13591053251318475] Babakazo P Bosonkie M Mafuta E , et al. (2022) Common breastfeeding problems experienced by lactating mothers during the first six months in Kinshasa. Plos One 17(10): e0275477. 10.1371/journal.pone.0275477 PMC9555666 36223384

[bibr2-13591053251318475] Bandura A (1986) Social Foundations of Thought and Action: A Social Cognitive Theory. Englewood Cliffs, NJ: Prentice-Hall.

[bibr3-13591053251318475] Baño-Piñero I Martínez-Roche ME Canteras-Jordana M , et al. (2018) Impact of support networks for breastfeeding: A multicentre study. Women and Birth 31(4): e239–e244. 10.1016/j.wombi.2017.10.002 29030022

[bibr4-13591053251318475] Bargh JA McKenna KYA Fitzsimons GM (2002) Can you see the real me? Activation and expression of the “true self” on the Internet. Journal of Social Issues 58(1): 33–48.

[bibr5-13591053251318475] Barnes JA (1969) Networks and political process. In: Mitchell JA (ed.) Social Networks in Urban Situations: Analysis of Personal Relationships in Central African Towns. Manchester, England: Manchester University Press, pp.51–76.

[bibr6-13591053251318475] Bennett GG Glasgow RE (2009) The delivery of public health interventions via the Internet: Actualizing their potential. Annual Review of Public Health 30(1): 273–292. 10.1146/annurev.publhealth.031308.100235 19296777

[bibr7-13591053251318475] Bratland KM Wien C Sandanger TM (2024) Exploring online health information seeking behaviour (OHISB) among young adults: A scoping review protocol. BMJ Open 14(1): e074894. 10.1136/bmjopen-2023-074894 PMC10828883 38296280

[bibr8-13591053251318475] Braun V Clarke V (2006) Using thematic analysis in psychology. Qualitative Research in Psychology 3(2): 77–101.

[bibr9-13591053251318475] Bridges N Howell G Schmied V (2018) Exploring breastfeeding support on social media. International Breastfeeding Journal 13(1): 22. 29983727 10.1186/s13006-018-0166-9 PMC6003082

[bibr10-13591053251318475] Brockway M Benzies K Hayden KA (2017) Interventions to improve breastfeeding self-efficacy and resultant breastfeeding rates: A systematic review and meta-analysis. Journal of Human Lactation 33(3): 486–499. 28644764 10.1177/0890334417707957

[bibr11-13591053251318475] Cambridge Dictionary (2024) Digital Age. Available at: https://dictionary.cambridge.org/dictionary/english/digital-age (accessed 7 March 2024).

[bibr12-13591053251318475] Cassel J (1976) The contribution of the social environment to host resistance: The fourth wade Hampton frost lecture. American Journal of Epidemiology 104(2): 798–123. 10.1093/oxfordjournals.aje.a112281 782233

[bibr13-13591053251318475] Centers for Disease Control and Prevention [CDC] (2024, October 23) What to expect while breastfeeding. Available at: https://www.cdc.gov/infant-?toddler-nutrition/breastfeeding/what-to-expect-whilebreastfeeding.html#:~:text=Pain%20while%20breastfeeding,milk%20(plugged%20milk%20duc)

[bibr14-13591053251318475] Chen J Wang Y (2021) Social media use for health purposes: Systematic review. Journal of Medical Internet Research 23(5): e17917. 10.2196/17917 PMC8156131 33978589

[bibr15-13591053251318475] Chivers BR Garad RM Moran LJ , et al. (2021) Support seeking in the postpartum period: Content analysis of posts in web-based parenting discussion groups. Journal of Medical Internet Research 23(7): e26600. 10.2196/26600 PMC8323017 34264198

[bibr16-13591053251318475] C.S. Mott Children’s Hospital (2023) Sharing on Parenting: Getting Advice Through Social Media. Available at: https://mottpoll.org/sites/default/files/documents/112023_Sharenting.pdf

[bibr17-13591053251318475] Davidson EL Ollerton RL (2020) Partner behaviours improving breastfeeding outcomes: An integrative review. Women and Birth 33(1): e15–e23. 10.1016/j.wombi.2019.05.010 31196832

[bibr18-13591053251318475] Dennis CL (1999) Theoretical underpinnings of breastfeeding confidence: A self-efficacy framework. Journal of Human Lactation 15(3): 195–201. 10.1177/089033449901500303 10578797

[bibr19-13591053251318475] Dieterich CM Felice JP O'Sullivan E , et al. (2013) Breastfeeding and health outcomes for the mother-infant dyad. Pediatric Clinics of North America 60(1): 31–48. 23178059 10.1016/j.pcl.2012.09.010 PMC3508512

[bibr20-13591053251318475] Dixon SJ (2024) Social Media – Statistics & Facts. Available at: https://www.statista.com/topics/1164/social-networks/#topicOverview

[bibr21-13591053251318475] Donelle L Hall J Hiebert B , et al. (2021) Investigation of digital technology use in the transition to parenting: Qualitative study. JMIR Pediatrics and Parenting 4(1): e25388. 10.2196/25388 PMC8078692 33595440

[bibr22-13591053251318475] Dutta-Bergman MJ (2004) Primary sources of health information: Comparisons in the domain of health attitudes, health cognitions, and health behaviors. Health Communication 16(3): 273–288. 15265751 10.1207/S15327027HC1603_1

[bibr23-13591053251318475] Ellis L Roberts L (2019) Exploring the use and quality of Internet discussion forums in pregnancy: A qualitative analysis. Birth 47(1): 153–161. 31583769 10.1111/birt.12459

[bibr24-13591053251318475] House JS Umberson D Landis KR (1988) Structures and processes of social support. Annual Review of Sociology 14(1): 293–318.

[bibr25-13591053251318475] Jackson KT O’Keefe-McCarthy S Mantler T (2019a) Moving toward a better understanding of the experience and measurement of breastfeeding-related pain. Journal of Psychosomatic Obstetrics and Gynaecology 40(4): 318–325. 30324846 10.1080/0167482X.2018.1518421

[bibr26-13591053251318475] Jackson KT Mantler T O'Keefe-McCarthy S (2019b) Women’s experiences of breastfeeding-related pain. MCN: The American Journal of Maternal Child Nursing 44(2): 66–72. 30688667 10.1097/NMC.0000000000000508

[bibr27-13591053251318475] Johnson JD (1997) Cancer-Related Information Seeking. Cresskill, NJ: Hampton Press.

[bibr28-13591053251318475] Kemp S (2024) Digital 2024: Global Overview Report. Available at: https://datareportal.com/reports/digital-2024-global-overview-report

[bibr29-13591053251318475] Lambert SD Loiselle CG (2007) Health information-seeking behavior. Qualitative Health Research 17(8): 1006–1019. 17928475 10.1177/1049732307305199

[bibr30-13591053251318475] Lincoln Y Guba E (1985) Naturalistic Inquiry. Newbury Park, CA: Sage.

[bibr31-13591053251318475] Lumivero (2024) NVivo (Version 14). Availavle at: www.lumivero.com

[bibr32-13591053251318475] Marcon AR Ravitsky V Caulfield T (2021) Discussing non-invasive prenatal testing on Reddit: The benefits, the concerns, and the comradery. Prenatal Diagnosis 41(1): 100–110. 33058217 10.1002/pd.5841

[bibr33-13591053251318475] Moorhead SA Hazlett DE Harrison L , et al. (2013) A new dimension of health care: Systematic review of the uses, benefits, and limitations of social media for health communication. Journal of Medical Internet Research 15(4): e85. 10.2196/jmir.1933 PMC3636326 23615206

[bibr34-13591053251318475] Morse H Brown A (2022) The benefits, challenges and impacts of accessing social media group support for breastfeeding: A systematic review. Maternal and Child Nutrition 18(4): e13399. 10.1111/mcn.13399 PMC9480914 35821651

[bibr35-13591053251318475] Pew Research Center (2023) Social Media Fact Sheet. Availavle at: https://www.pewresearch.org/internet/fact-sheet/social-media/?tabId=tab-0ec23460-3241-4a1f-89bc-0c27fb641936

[bibr36-13591053251318475] Pirraglia PA Kravitz RL (2013) Social media: New opportunities, new ethical concerns. Journal of General Internal Medicine 28(2): 165–166. 23225258 10.1007/s11606-012-2288-x PMC3614145

[bibr37-13591053251318475] Powell J Inglis N Ronnie J , et al. (2011) The characteristics and motivations of online health information seekers: Cross-sectional survey and qualitative interview study. Journal of Medical Internet Research 13(1): e20. 10.2196/jmir.1600 PMC3221342 21345783

[bibr38-13591053251318475] Record RA Silberman WR Santiago JE , et al. (2018) I sought it, I Reddit: Examining health information engagement behaviors among Reddit users. Journal of Health Communication 23(5): 470–476. 29718799 10.1080/10810730.2018.1465493

[bibr39-13591053251318475] Reddit (2016) The Social Media Revolution: An Economic Encyclopedia of Friending, Following, Texting, and Connecting. Greenword Press: Westport, Connecticut. pp.299–300.

[bibr40-13591053251318475] Reddit (2023) Reddiquette. Available at: https://support.reddithelp.com/hc/en-us/articles/205926439-Reddiquette

[bibr41-13591053251318475] Regan S Brown A (2019) Experiences of online breastfeeding support: Support and reassurance versus judgement and misinformation. Maternal and Child Nutrition 15(4): e12874. 10.1111/mcn.12874 PMC6859975 31299699

[bibr42-13591053251318475] Skelton KR Evans R LaChenaye J , et al. (2018) Exploring social media group use among breastfeeding mothers: Qualitative analysis. JMIR Pediatrics and Parenting 1(2): e11344. 10.2196/11344 PMC6715055 31518305

[bibr43-13591053251318475] Suler J (2004) The online disinhibition effect. CyberPsychology & Behavior 7(3): 321–326. 15257832 10.1089/1094931041291295

[bibr44-13591053251318475] Today’s Parent (2018) “I Need My People.” How Social Media is Actually Helping Canadians Become Better Parents. Available at: https://www.todaysparent.com/family/parenting/i-need-my-people-how-social-media-is-actually-helping-canadians-become-better-parents/

[bibr45-13591053251318475] Ventola CL (2014) Social media and health care professionals: Benefits, risks, and best practices. Pharmacy and Therapeutics 39(7): 491–520. 25083128 PMC4103576

[bibr46-13591053251318475] Zimmerman DR Kaplan M Shoob H , et al. (2022) Breastfeeding challenges and support in a high initiation population. Israel Journal of Health Policy Research 11(1): 31. 36071536 10.1186/s13584-022-00538-5 PMC9449948

